# A Combination of Biochar–Mineral Complexes and Compost Improves Soil Bacterial Processes, Soil Quality, and Plant Properties

**DOI:** 10.3389/fmicb.2016.00372

**Published:** 2016-04-08

**Authors:** Jun Ye, Rui Zhang, Shaun Nielsen, Stephen D. Joseph, Danfeng Huang, Torsten Thomas

**Affiliations:** ^1^Centre for Marine Bio-Innovation & School of Biotechnology and Biomolecular Sciences, The University of New South WalesSydney, NSW, Australia; ^2^School of Agriculture and Biology, Shanghai Jiao Tong UniversityShanghai, China; ^3^School of Materials Science and Engineering, The University of New South WalesSydney, NSW, Australia

**Keywords:** biochar, compost, soil microbiology, plant productivity, soil microbial communities

## Abstract

Organic farming avoids the use of synthetic fertilizers and promises food production with minimal environmental impact, however this farming practice does not often result in the same productivity as conventional farming. In recent years, biochar has received increasing attention as an agricultural amendment and by coating it with minerals to form biochar–mineral complex (BMC) carbon retention and nutrient availability can be improved. However, little is known about the potential of BMC in improving organic farming. We therefore investigated here how soil, bacterial and plant properties respond to a combined treatment of BMC and an organic fertilizer, i.e., a compost based on poultry manure. In a pakchoi pot trial, BMC and compost showed synergistic effects on soil properties, and specifically by increasing nitrate content. Soil nitrate has been previously observed to increase leaf size and we correspondingly saw an increase in the surface area of pakchoi leaves under the combined treatment of BMC and composted chicken manure. The increase in soil nitrate was also correlated with an enrichment of bacterial nitrifiers due to BMC. Additionally, we observed that the bacteria present in the compost treatment had a high turnover, which likely facilitated organic matter degradation and a reduction of potential pathogens derived from the manure. Overall our results demonstrate that a combination of BMC and compost can stimulate microbial process in organic farming that result in better vegetable production and improved soil properties for sustainable farming.

## Introduction

In order to balance conservation of natural resources and food production, contemporary agriculture and horticulture rely heavily on soil amendments to ensure and improve soil quality and plant yields (Ramankutty and Rhemtulla, 2012). One such amendment is biochar, a carbon-rich, solid material derived from the thermal processing of organic feedstocks in an air-starved environment (Chan et al., 2008). Traditional use of biochar has been linked to the high fertility of Amazonian Dark Earths (ADE; Glaser et al., 2000) and recent work has revealed that ADE biochar can interact with surrounding organic matter, ash, clay, and fragments of bones during weathering to form mineral-aggregates (Chia et al., 2012). Based on these findings, biochar has been recently torrefied (< 240°C) with ground rocks, clays, and other minerals to form biochar–mineral complexes (BMC), which mimic the biochar mineral-aggregates found in ADE (Chia et al., 2010, 2014). Furthermore, the production process of BMCs can be controlled to conserve up to 73% of the initial total nitrogen present in the organic feedstock as the nitrogen can be incorporated into heterocyclic structures, which provide a relatively stable and slow-release form of nitrogen (Lin et al., 2013). BMC can also have high concentrations of exchangeable cations and mean residence time of ~150 years (Chia et al., 2014) as mineral additives significantly increased the stability of the organic components in the biochar (Li et al., 2014).

Although BMC has the potential to increase nutrient availability and sequester carbon for long periods of time, its impact on nutrient cycling in soil as well as its benefits for plant growth are not well-understood. Interestingly, the carbon-rich ADE have been shown to contain distinct microbial communities as well as higher microbial richness and biomass when compared to adjacent soil (O'Neill et al., 2009; Grossman et al., 2010). BMC has also recently been shown at low application rates (1–5 t ha^−1^) to alter the bacterial communities in contemporary soils when compared to traditional fertilization (Nielsen et al., 2014). Given the central role of bacteria in nutrient cycling in soil and their many positive and negative interactions with plants, the effect of BMC in agricultural situations might be partially or indirectly mediated via its impact on the soil microbiota itself.

Another common organic soil amendment is compost, which is mainly derived from easily degradable animal manure or green waste and frequently used in organic farming (Rigby and Cáceres, 2001). Compost generally is not only abundant in nutrients, but also harbors vast amounts of microorganisms, whose compositions largely depends on the source material (Sun et al., 2015). For instance, compost based on animal manure contains bacteria originated from the guts of animals (Unc and Goss, 2004). However, whether the introduction of these microorganisms from compost affect nutrient processes in soil is poorly understood (Hartmann et al., 2015). While organic fertilizers such as compost have less negative consequence for the soil ecosystem than synthetic fertilizers, there are on their own not quite competitive in terms of plant yield under most agricultural and horticultural settings (Seufert et al., 2012). This might be due to the compost's nutrients being either too slowly released or of a suboptimal composition to support the plant's nutrient demands (Berry et al., 2002).

Given the ability of BMC to influence microbial communities and processes in soil (see above), we hypothesized here that BMC can be used to alter nutrient conversion and availability from compost and that this can lead to better plant growth. To test this hypothesis we analyzed plant yields, soil properties, and bacterial communities under treatments of either BMC or compost alone or in their combination as well as in comparison to an unamended control treatment. As a study system, we chose pakchoi, a major vegetable grown in China in intensive horticultural settings.

## Materials and methods

### Experimental material

The soil used in this experiment was collected at an experimental station of Shanghai Jiao Tong University located in Chongming Island, Shanghai, China (31°48′N, 121°74′E). This collection site had vegetable farming from 2008 to 2011. In 2011 the soil was amended with compost with no further activity until the sampling for the experiment. Bulk soil (0–20 cm depth) was collected in July 2014 after removing the top vegetation, which was mainly grass. The soil is characterized as light loam. The soil was sieved through 5 mm mesh and homogenized in the laboratory.

Fresh chicken manure provided by a suburban poultry farm was composted for 30 days and used as compost in this study. Biochar was produced from jarrah wood (Simcoa Pty. Ltd., Bunbury, Western Australia) in a vertical retort with a residence time of ~12 h and a maximum temperature of 600°C. This biochar was activated with phosphoric acid and mixed with high iron bearing kaolinitic clay (30%, w/w), chicken manure (30%, w/w), rock phosphate, basalt dust illmenite, and dolomite. This mixture was torrefied at 220°C for 3 h to produce BMC. After pyrolysis the BMC was stored aseptically in ziplock bags. The basic physicochemical properties of soil, compost, and BMC are shown in Table 1.

**Table 1:** **Basic properties of soil, compost and biochar–mineral complex (BMC) used in this study**.

	**Soil**	**Compost**	**BMC**
pH (CaCl_2_)	8.17	9.22	6.98
EC (μs cm^−1^)	476.00	11,576.67	1420.67
TC (mg g^−1^)	17.22	191.91	265.74
TN (mg g^−1^)	1.12	27.12	10.01
Calcium (mg g^−1^)	28.28	134.03	68.00
Phosphorus (mg g^−1^)	0.84	27.98	26.00
Magnesium (mg g^−1^)	14.19	14.56	14.00
Iron (mg g^−1^)	35.34	4.72	7.00
Potassium (mg g^−1^)	20.14	23.12	4.70
Sulfur (mg g^−1^)	0.55	5.17	4.60
Aluminum (mg g^−1^)	68.34	9.08	2.10
Sodium (mg g^−1^)	10.85	6.96	1.80
Zinc (mg g^−1^)	0.12	0.82	0.13
Copper (mg g^−1^)	0.04	0.06	0.03

### Pot trial

A pot trial was carried out with a full factorial design including the following four treatments: (1) no additions (CK), (2) 0.1% (w/w) BMC (equivalent to 1.5 t ha^−1^), (3) 1.9% (w/w) compost (treatment CO, equivalent to 28.5 t ha^−1^), and (4) 0.1% (w/w) BMC and 1.9% (w/w) compost (treatment BMCO). Soil and amendments for each treatment were homogenized on a sterile working bench and three random samples were taken from each mixture for microbial community analysis at day 1. Treatment mixtures were then divided into 15 replicate pots (top diameter 10 cm, bottom diameter 9 cm, height 10 cm, 500 g pot^−1^).

Twenty aseptic seeds of the commercial variety of pakchoi (*Brassica rapa* L. ssp. *chinensis*) named “Huawang” were sown into each pot. The pots were then randomly placed in an array (with 5 cm intervals) within a climate-controlled chamber. The chamber was configured to a day temperature of 20°C, a night temperature of 18°C and a photoperiod of 14 h with a light intensity of 300 μmol m^−2^ s^−1^ photosynthetically active radiation. Irrigation was applied twice per week. To achieve a realistic planting density, only six uniform seedlings were kept in each pot after seedlings reached the stage of one leaf and one bud (about 6 days). No additional fertilizer was added during the cultivation.

At 40 days after planting, the pakchoi were harvested and the total soil was collected from each pot. The soil and pakchoi of three random pots per treatment were pooled resulting in five replicate pools to be used for further analysis. The soil was homogenized and passed through a 2 mm sieve to remove roots and debris. Plant and soil samples were divided into two subsamples. Plant subsamples were used for either physical or chemical analysis, whereas soil subsamples were used for chemical or microbial community analysis. All samples were placed in sterile bags on dry ice at the time of sampling, and then stored either at 4 or −80°C, as required for downstream analysis.

### Analysis of edaphic and plant properties

Soil moisture was determined by drying fresh soil at 60°C for 4 days. Soil pH was measured by suspending dried soil in a 0.01 M calcium chloride solution (2:1, w/v; Jones, 2001). Electrical conductivity (EC) was measured for soil suspended in deionized water (5:1, w/v; Corwin and Lesch, 2005). Total soil carbon (TC) and total soil nitrogen (TN) were measured using a vario EL III elemental analyzer (Elementar, Germany). Soil organic carbon (OC) was measured by a standard chromic acid titration method (Walkley and Black, 1934). Soil available phosphorus (AP) was analyzed in extracts with 0.5 M sodium bicarbonate (1:20 w/v) using a spectrophotometric method (Olsen, 1954). Soil available potassium (AK) was analyzed in extracts with 1 M ammonium acetate (1:10, w/v) using flame atomic emission spectroscopy (Brown, 1998). Automated discrete analyzer (Smartchem, France) was used to analyze total soluble nitrogen (TSN), nitrate (${\text{NO}}_{3}^{-}$), and ammonium (${\text{NH}}_{4}^{+}$) in 2 M KCl soil extracts (1:10, w/v).

The fresh weight, dry weight and water content of the pakchoi were measured by comparing mass before and after drying at 105°C for 30 min and at 60°C to constant weight. The largest leaf of each pakchoi plant was chosen for leaf area and height measurement. Leaves were scanned using a Perfection V700 scanner (Epson, USA) and the resulting image was processed using the WinRhizo software (Regent Instruments Inc., Canada) to determine the leaf length and surface area. Fresh pakchoi was used for chlorophyll, soluble protein, soluble saccharides, and nitrate measurement with standard methods (Li et al., 2000).

### Bacterial analysis

Soil samples from the beginning (Day 1) and the end (Day 40) of the cultivation period were subjected to total DNA extraction and sequencing of the 16S rRNA gene. Total DNA was extracted from 0.5 g sieved soil using the PowerSoil DNA Isolation kit (MO BIO Laboratories Inc., USA) according to the manufacturer's instruction. DNA quality and concentrations were determined using a Nanodrop 1000 (Thermo Fisher Scientific Inc. USA). The V1–V3 regions of the 16S rRNA gene were amplified using barcoded primers 27F (5′-AGAGTTTGATCMTGGCTCAG-3′) and 519R (5′-GWATTACCGCGGCKGCTG-3′) that target conserved sequences found in bacteria. Amplicons from each PCR sample were normalized to equimolar amounts and sequenced on a MiSeq platform (Illumina Inc., USA) at the Ramaciotti Centre for Genomics (University of New South Wales, Australia). The sequencing data has been deposited at the National Centre for Biotechnology Information under BioProject accession no. PRJNA297134.

16S rRNA gene sequencing data was processed using the Mothur pipeline (Kozich et al., 2013). Sequence reads were discarded on a per-contig basis, if sequences contained an N base or had more than eight homopolymers. Trimmed sequences were aligned using the Silva 16S rRNA gene reference alignment (Pruesse et al., 2007), screened to include only overlapping regions based on alignment positions (start = 1045, end = 13,126), pre-clustered (diffs = 1) and checked for chimeras using UCHIME (Edgar et al., 2011). Singletons were removed from the dataset to reduce noise associated with sequencing errors caused by the sequencing process (Reeder and Knight, 2009). Finally, the number of sequences was normalized by randomly subsampling each sample to the lowest number of sequence counts present in all samples that were compared (i.e., 5793 counts when all samples were compared or 11,076 when only day 40 samples were compared). The taxonomic assignment of the sequences were performed using the Greengenes database (May, 2013 version) with 60% confidence cut-off and clustered into operational taxonomic units (OTU) at 97% identity with consensus taxonomy. Any sequences that were classified as “Mitochondria,” “Eukaryotic,” or “Chloroplast” as well as any sequences of unknown origin were removed from the dataset. The resulting quality-filtered dataset was used in subsequent data analysis.

### Statistical analysis

Experimental treatments were factorized into cultivation time (CT), compost (CO), and BMC in the following analysis. As plants grew in small pots, any temporal variation seen in the soil could be due to time and/or the introduction of the root system. Hence, the factor of CT includes the effect of both time and root input. All factors had two levels, that is day 1 and day 40 for CT, with and without additives for CO and BMC.

To analyze the overall effects of CO and BMC, we compared edaphic and plant variables using both multivariate and univariate methods. For the multivariate analysis, unconstrained principal coordinates analysis (PCoA) was applied to the Euclidean distance matrix generated from normalized soil and plant data. Two-way permutational analysis of variance (PERMANOVA) was used to test the effect of each factor. For the univariate analysis, edaphic variables, and plant properties were subjected to a two-way analysis of variance (ANOVA). In addition, the net increments of TC and TN in each treatment were calculated as the average difference between treatments and control and considering the inherent carbon and nitrogen introduced through the treatments (see Table 1).

Chao1 and Simpson evenness indices were calculated as α-diversity estimators for bacterial community structure, while β-diversity was compared using the Bray–Curtis similarity coefficient calculated on square-root transformed relative abundances of OTUs. Differences between Chao1 and Simpson evenness indices were analyzed using a three-way ANOVA (CT × CO × BMC). The Bray–Curtis similarity coefficient matrix was visualized using unconstrained PCoA. Three-way PERMANOVA was applied to the Bray–Curtis similarity matrix to test the significance levels of differences between experimental factors and to explain the contribution of each factor to the total variance. Since the discriminative power of permutation-based analyses for pairwise comparisons of three and five replicates was limited by 10 and 126 unique permutations, respectively, we obtained pairwise comparisons of within-group to between-group Bray–Curtis similarities using the non-parametric Wilcoxon rank sum test (Wilcoxon, 1945). Given that significant differences detected by PERMANOVA could be due to similarity, dispersion or both (Clarke and Warwick, 2001), the homogeneity of dispersion test (PERMDISP) was performed to examine the differences in multivariate dispersion between groups.

Distance-based linear models (DISTLM) were used to explore the relationship between edaphic variables and bacterial β-diversity (Mcardle and Anderson, 2001). A marginal test that examines each variable exclusively and a sequential test that examines each variable after fitting all variables in one model were first applied to explore the predictive potential of variables. Forward selection procedure and the adjusted *R*^2^ selection criterion were used in sequential tests. A permutation-based test was further implemented to obtain the significance *P*-value for these relations. The model that captures the most variance in the soil's bacterial community was subsequently calculated by the BEST selection procedure and the adjusted *R*^2^ selection criterion. Distance-based redundancy analysis (dbRDA) with multiple partial correlations was performed to visualize the model given by BEST selection.

The OTUs exhibited significant differences between treatments at each cultivation time were detected using a two-way ANOVA (CO × BMC) within STAMP (Parks et al., 2014). The association strength of each OTU with target treatments was determined using indicator species analysis based on point biserial correlation coefficients (De Cáceres and Legendre, 2009). As OTUs can be shared between treatments, all possible combinations of *a priori* groups of treatments were tested (De Cáceres et al., 2010). The analysis was performed using the multipatt function in the indicspecies package of R. OTUs that have < 10 sequence counts were excluded, as they have limited indicator potential. The distribution and association strength of OTUs that have relative abundances over 1% in at least one sample were visualized using pheatmap package in R.

PERMANOVA, PERMDISP, DISTLM, BEST, and dbRDA were performed in PRIMER v6 package (Anderson et al., 2008). All permutation-based tests were conducted with 10^5^ permutations. *P*-value adjustment was applied for multiple comparisons using Benjamini–Hochberg FDR procedure in R with p.adjust function. Figures were generated in R with the ggplot2 package (Wickham, 2009).

## Results

### The effect of compost and biochar–mineral complex on edaphic and plant variables

**Figure 1:**
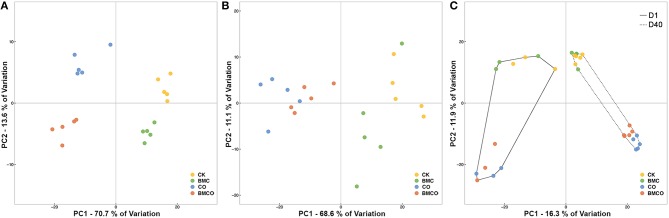
**Unconstrained principal coordinates analysis (PCoA) plots illustrating the similarities in individual samples based on: (A) Euclidean distances of edaphic variables, (B) Euclidean distance of plant variables, (C) Bray–Curtis distance of bacterial communities**. Edaphic and plant variables were normalized before calculating the Euclidean distance. Relative abundance of bacterial communities was square root transformed before calculating the Bray–Curtis distance.

The edaphic parameters for all four treatments (CK, BMC, CO, BMCO) were clearly grouped after 40 days of pakchoi cultivation (Figure 1A), whereas plant parameters only showed a clear separation between treatments with and without compost (i.e., CK and BMC vs. CO and BMCO, Figure 1B). Although, there were prominent effects on soil and plant variables associated with CO and BMC, significant interactions were observed in both edaphic (*P* = 0.001) and plant (*P* < 0.001) parameters (Table 2). However, the interaction effects were small compared to the variance component of CO, which was at least three times greater than that of the BMC or CO × BMC terms. Moreover, pairwise comparisons generally showed that the combination of BMC and CO (i.e., treatment BMCO) had larger effects when compared to BMC or CO treatments alone. Taken together, this statistical analysis indicates that CO was the main factor in changing soil and plant parameters and that BMC plays a different role when combined with compost than on its own.

**Table 2:** **Effect of treatments (CK: control, BMC: biochar–mineral complex, CO: compost, BMCO: BMC + CO) on Euclidean distance of samples**.

**Main test**	**Soil**			**Plant**		
	***F*_(1, 16)_**	***P***	***VC***	***F*_(1, 16)_**	***P***	***VC***
CO	**57.02**	**<0.001**	**17.80**	**32.65**	**<0.001**	**9.58**
BMC	**12.84**	**<0.001**	**3.76**	**2.18**	**0.048**	**0.36**
CO × BMC	**3.82**	**0.001**	**1.79**	**5.67**	**<0.001**	**2.83**
**Pairwise test**	***t***	***P**_*adjust*_*	***DIS***	***t***	***P**_*adjust*_*	***DIS***
CK_D40 vs. BMC_D40	**2.63**	**0.007**	**3.36**	1.63	0.056	2.97
CO_D40 vs. BMCO_D40	**3.08**	**0.008**	**4.15**	**2.36**	**0.010**	**3.02**
CK_D40 vs. CO_D40	**4.90**	**0.008**	**6.39**	**5.74**	**0.010**	**6.16**
BMC_D40 vs. BMCO_D40	**6.26**	**0.008**	**6.73**	**2.94**	**0.010**	**4.04**
CK_D40 vs. BMCO_D40	**6.80**	**0.008**	**8.00**	**5.06**	**0.010**	**4.83**
BMC_D40 vs. CO_D40	**4.82**	**0.008**	**5.80**	**3.65**	**0.010**	**5.16**

*Euclidean distances were calculated based on normalized soil and plant variables, respectively. Effects of main factors (i.e., CO and BMC) and their interaction (i.e., CO × BMC) were assessed by multivariate permutational analysis of variance (PERMANOVA) with 10^5^ permutations. Values represent the pseudo-F ratio with degrees of freedom and denominator in brackets [F_(1, 16)_], the permutation-based level of significance (P), the variance component (VC), the pairwise t-statistic (t), and the average distance between-groups (DIS). Multiple comparison adjustment was applied using Benjamini–Hochberg procedure. Values at P_adjust_ < 0.05 are shown in bold*.

Univariate analysis showed that CO had a significant impact on all variables, except soil pH, while BMC displayed effects on humidity, TN, TSN, AK, and leaf area (Table S1). Pairwise comparisons at the treatments level further showed that soil humidity significantly decreased from 16.61 ± 3.19 to 12.19 ± 1.09% when CK was compared to CO (*P* = 0.025, Table 3). However, the addition of BMC increased soil humidity both alone (19.32 ± 2.66%) and in combination with compost (i.e., treatment BMCO, 14.71 ± 0.59%) as compared with the CK and CO treatments, respectively. BMC and CO also increased soil EC by 6 and 26%, respectively. There were no detectable changes in soil pH, despite the fact that the compost (pH = 9.22) and BMC (pH = 6.98) materials themselves had pH values substantial above and below that of the soil (pH = 8.17, Table 1). As would be expect, all nutrient values significantly increased in the soil with the addition of compost. An additional increase of certain nutrient values was observed when compost was combined with BMC (i.e., treatment BMCO). Specifically, BMC interacted with compost synergistically to further increase TC, TN, OC, ${\text{NO}}_{3}^{-}$, and AK, while BMC on its own caused only small changes compared to the CK treatment. BMC also caused a major increase in TSN either on its own or in an additive manner with compost. In contrast, BMC decreased AP content in soil, and this effect was even more pronounced in the BMCO treatment.

**Table 3:** **Average edaphic and plant parameters (mean ± *s.e*.; *n* = 5) for each treatments**.

	**CK**	**BMC**	**CO**	**BMCO**
**SOIL**				
Humidity (%, m/m)	16.61 ± 3.19ab	19.32 ± 2.66a	12.19 ± 1.09c	14.71 ± 0.59bc
pH (CaCl_2_)	8.21 ± 0.06a	8.14 ± 0.02a	8.14 ± 0.04a	8.14 ± 0.02a
EC (μs cm^−1^)	534.2 ± 20.95c	565.4 ± 8.73b	673.2 ± 19.29a	661.6 ± 11.61a
TC (g kg^−1^)	16.96 ± 0.18c	17.24 ± 0.22c	22.43 ± 2.52b	26.38 ± 1.73a
TN (g kg^−1^)	1.14 ± 0.09c	1.18 ± 0.09c	1.85 ± 0.29b	2.18 ± 0.15a
OC (g kg^−1^)	9.54 ± 0.16c	10.04 ± 0.41c	13.63 ± 1.62b	17.05 ± 1.25a
TSN (mg kg^−1^)	36.99 ± 1.54c	64.66 ± 4.48b	62.41 ± 1.57b	90.44 ± 5.60a
${\text{NO}}_{3}^{-}$ (mg kg^−1^)	1.96 ± 0.53c	3.08 ± 0.75bc	4.02 ± 1.25b	8.81 ± 2.34a
NH_4_ (mg kg^−1^)	3.66 ± 0.35b	4.72 ± 0.62b	7.01 ± 0.45a	6.94 ± 1.10a
AP (mg kg^−1^)	10.24 ± 0.63bc	5.44 ± 0.50c	37.81 ± 8.93a	15.96 ± 2.41b
AK (mg kg^−1^)	133.52 ± 5.08c	152.36 ± 10.2c	347.33 ± 35.36b	384.06 ± 12.99a
**PLANT**				
Height (cm)	8.86 ± 0.17c	9.26 ± 0.62c	14.5 ± 0.95a	13.35 ± 0.73a
Fresh weight (g)	1.72 ± 0.27c	1.92 ± 0.30c	9.3 ± 1.55a	7.2 ± 1.41b
Dry weight (g)	0.02 ± 0.01c	0.05 ± 0.03c	0.37 ± 0.10a	0.2 ± 0.08b
Leaf area (cm^2^)	2.23 ± 0.60c	2.41 ± 0.46c	3.39 ± 0.55bc	4.81 ± 0.98a
Water content (%, m/m)	98.59 ± 0.30a	97.24 ± 1.44ab	96.11 ± 0.51b	97.26 ± 0.61ab
Chlorophyll (mg g^−1^)	1.57 ± 0.34c	2.03 ± 0.13bc	3.09 ± 0.67a	2.76 ± 0.25ab
Soluble protein (mg g^−1^)	29.83 ± 4.90b	29.34 ± 3.12b	38.23 ± 4.35a	32.43 ± 2.03ab
Soluble saccharides (mg g^−1^)	9.71 ± 1.15a	6.72 ± 6.67ab	3.33 ± 1.12b	3.79 ± 1.05ab
Nitrate (μg g^−1^)	283.41 ± 41.14a	213.91 ± 18.45b	146.3 ± 35.68c	177.9 ± 30.00bc

*Low case letters indicates significant differences assessed by ANOVA at P < 0.05*.

As for plant parameters, the CO and BMCO treatments resulted in a substantial increase in yield (in terms of height and weight) for pakchoi relative to the CK and BMC treatments. Surprisingly though, the combination of BMC and compost (treatment BMCO) resulted in a further increase in leaf area reaching values of up to 4.81 ± 0.98 cm^2^, which was not achieved by adding BMC or compost alone (Table 3). The addition of compost alone caused a decrease in soluble saccharides and nitrate, yet an increase in soluble protein and chlorophyll, when compared to the control. The addition of BMC to soil alone or soil amended with compost had little impact on the chlorophyll, soluble saccharides and protein content. However, BMC notably decreased the nitrate in pakchoi when used on its own.

### The effect of compost and biochar–mineral complex on bacterial diversity

Given the results above, the addition of compost and BMC (either alone or combination) had clear additive and synergistic effects on the nutrient content of the soil. We hypothesized that this might be due to changes in metabolic processes occurring in the soil and therefore investigated the composition of its bacterial community. At total of 1,174,380 high-quality 16S rRNA sequences were generated from 32 samples covering three replicates of day 1 and five replicates of day 40 soil samples for each of the four treatments. After subsampling and clustering, 10,509 operational taxonomic units (OTUs) at 97% identity (roughly corresponding to species) were obtained, with the number of OTUs ranging from 519 to 1940 per sample. The Good's coverage for the observed OTUs was 89.2 ± 5.5% at a sequencing depth of 5793 reads, which indicates good sampling of the community.

The addition of compost (treatments CO and BMCO) significantly decreased the bacterial evenness in samples of day 1 (*P* < 0.001, Table S2). BMC caused a slight decrease in the bacterial richness at the beginning of the experiment, while CO had no impact on microbial richness (*P* = 0.604, Table S2). Interestingly, bacterial richness and evenness of all treatments reached the same level after 40 days of cultivation, with the richness being almost doubled compared to the beginning of the pot trial (*P* < 0.001).

The composition of bacterial communities was largely influenced by the factors CT and CO (Figure 1C). The first PCoA axis separated samples based on the two sampling times (i.e., Day 1 and 40), whereas the influence of the compost was shown along the second PCoA axis. The replicate samples at day 1 appeared to be more divergent than those at day 40, which was supported by a PERMDISP analysis that showed that the average distance to the treatments centroid for day 1 samples was 27.6% larger than for day 40 samples (*P* < 0.001, Table S3). In addition, the dispersion associated with CO was negligible (*P* = 0.962, Table S3), suggesting that the differences in bacterial β-diversity between groups with (CO and BMCO) and without (CK and BMC) compost were mainly due to the dissimilarity between groups rather than the dispersion within groups.

The PERMANOVA main test suggested a large effect on β-diversity associated with the interaction between the factors CO and CT (*VC* = 19.77, *P* < 0.001) and a smaller effect associated with the interaction between the factors BMC and CT (*VC* = 7.74, *P* = 0.012). Pairwise comparison within levels of CT showed that CO preserved a significant effect over time (Table 4). This supports the notion that compost delivered distinct bacterial communities into the soil at the beginning of the pot trial and significantly transformed the bacterial communities originally in soil during the cultivation period. The effect of BMC was relatively small compared to the CO (Table 4). Nevertheless, there was still an apparent difference between the CO and BMCO treatment at day 40 (Figure 1C), which indicates additional change in the bacterial communities on top of those caused by compost.

**Table 4:** **Effect of treatments on bacterial β-diversity**.

**Main test**	***F*_(1, 25)_**	***P***	***VC***
CO	**5.10**	**<0.001**	**19.55**
BMC	**1.31**	**0.013**	**5.41**
CT	**6.18**	**<0.001**	**21.97**
CO × BMC	1.19	0.099	5.95
CO × CT	**3.10**	**<0.001**	**19.77**
BMC × CT	**1.32**	**0.012**	**7.74**
CO × BMC × CT	1.22	0.061	9.15
**Pairwise test**	***t***	***P**_*adjust*_*	***SIM***
CK_D1 vs. BMC_D1	1.02	0.864	31.02
CO_D1 vs. BMCO_D1	1.05	1.000	39.77
CK_D1 vs. CO_D1	**1.42**	**0.026**	**29.71**
BMC_D1 vs. BMCO_D1	**1.30**	**0.026**	**25.43**
CK_D1 vs. BMCO_D1	**1.36**	**<0.001**	**28.56**
BMC_D1 vs. CO_D1	**1.39**	**0.018**	**25.43**
CK_D40 vs. BMC_D40	1.02	0.760	49.59
CO_D40 vs. BMCO_D40	1.06	0.291	54.90
CK_D40 vs. CO_D40	**1.95**	**<0.001**	**41.97**
BMC_D40 vs. BMCO_D40	**1.71**	**<0.001**	**42.94**
CK_D40 vs. BMCO_D40	**1.73**	**<0.001**	**43.69**
BMC_D40 vs. CO_D40	**1.93**	**<0.001**	**41.07**
CK_D1 vs. CK_D40	**1.31**	**0.002**	**38.93**
BMC_D1 vs. BMC_D40	**1.38**	**0.003**	**32.64**
CO_D1 vs.CO_D40	**2.18**	**<0.001**	**30.94**
BMCO_D1 vs. BMCO_D40	**1.99**	**<0.001**	**29.75**

*Effects of main factors and their interactions were assessed by multivariate permutational analysis of variance (PERMANOVA) with 10^5^ permutations. Values represent the pseudo-F ratio with degrees of freedom and denominator in brackets [F_(1, 25)_], the permutation-based level of significance (P) and the variance component (VC). Pairwise test was performed based on levels of cultivation time. Values represent the t-statistic (t) and the average between-group Bray–Curtis similarity (SIM). Since the discriminative power of permutation-based analyses for pairwise comparisons of three replicates and five replicates was limited by 10 and 126 unique permutations, respectively, P-values were obtained by comparing within-group to between-group Bray–Curtis similarities using the Wilcoxon rank sum test instead. Multiple comparison adjustment was applied using Benjamini–Hochberg procedure. Values at P_adjust_ < 0.05 are shown in bold*.

### Links between edaphic variables and bacterial communities

Both soil parameters and bacterial communities were significantly influenced by the application of compost and BMC after 40 days (see Sections The Effect of Compost and Biochar-Mineral Complex on Edaphic and Plant Variables and The Effect of Compost and Biochar-Mineral Complex on Bacterial Diversity), and we therefore explored correlations between soil variables and microbial β-diversity using a distance-based linear regression method. Marginal tests showed that all variables (except pH) had significant correlations with bacterial communities (Table 5). Furthermore, because many edaphic variables may explain the same portion of variation in bacterial β-diversity, sequential tests were performed to examine the remaining variation conditional on other variables (Mcardle and Anderson, 2001). This revealed that all variables together explained 70.3% of the variance in bacterial β-diversity. EC made the strongest contribution by explaining 22.3% of the bacterial β-diversity variation (*P* < 0.05), humidity and C:N explained an additional 5.4% (*P* = 0.02) and 5.2% (*P* = 0.05), respectively. These variables constituted the BEST selection solution (i.e. the best fitted model). dbRDA analysis illustrated the adequacy of the model comprised of these three variables to predict patterns of bacterial β-diversity (Figure S1). Differences of bacterial β-diversity associated with CO were separated by EC and C:N, while differences associated with BMC were separated by soil humidity.

**Table 5:** **Distance-based linear modeling examining the relationship between edaphic variables and microbial communities**.

	**Marginal test**		**Sequential test**	
	***VC* (%)**	***P*_*adjust*_**	***VC* (%)**	***P*_*adjust*_**
EC	**22.3**	**<0.05**	**22.3**	**<0.05**
Humidity	**14.8**	**<0.05**	**5.4**	**0.02**
C:N	**19.4**	**<0.05**	**5.2**	**0.05**
AP	**15.8**	**<0.05**	4.6	0.27
OC	**18.0**	**<0.05**	4.6	0.25
AK	**22.3**	**<0.05**	4.5	0.36
TSN	**10.4**	**<0.05**	4.3	0.41
pH	6.5	0.15	4.2	0.46
NO_3_	**11.8**	**<0.05**	4.2	0.46
TC	**18.5**	**<0.05**	3.8	0.59
TN	**19.8**	**<0.05**	3.6	0.61
NH_4_	**19.3**	**<0.05**	3.5	0.65
Total			70.3	

*The marginal test examines the relationship of each edaphic variable on microbial communities individually, whereas the sequential test examines the relationship by sequentially fitting all variables into the most parsimonious model. The sequential test was conducted using a forward selection procedure and the adjusted R^2^ selection criterion. VC represents for the explained variance component and P_adjust_ represents the Benjamini–Hochberg adjusted level of significance. Values at P_adjust_ < 0.05 are shown in bold*.

### Taxonomic composition and treatment-associated taxa

**Figure 2:**
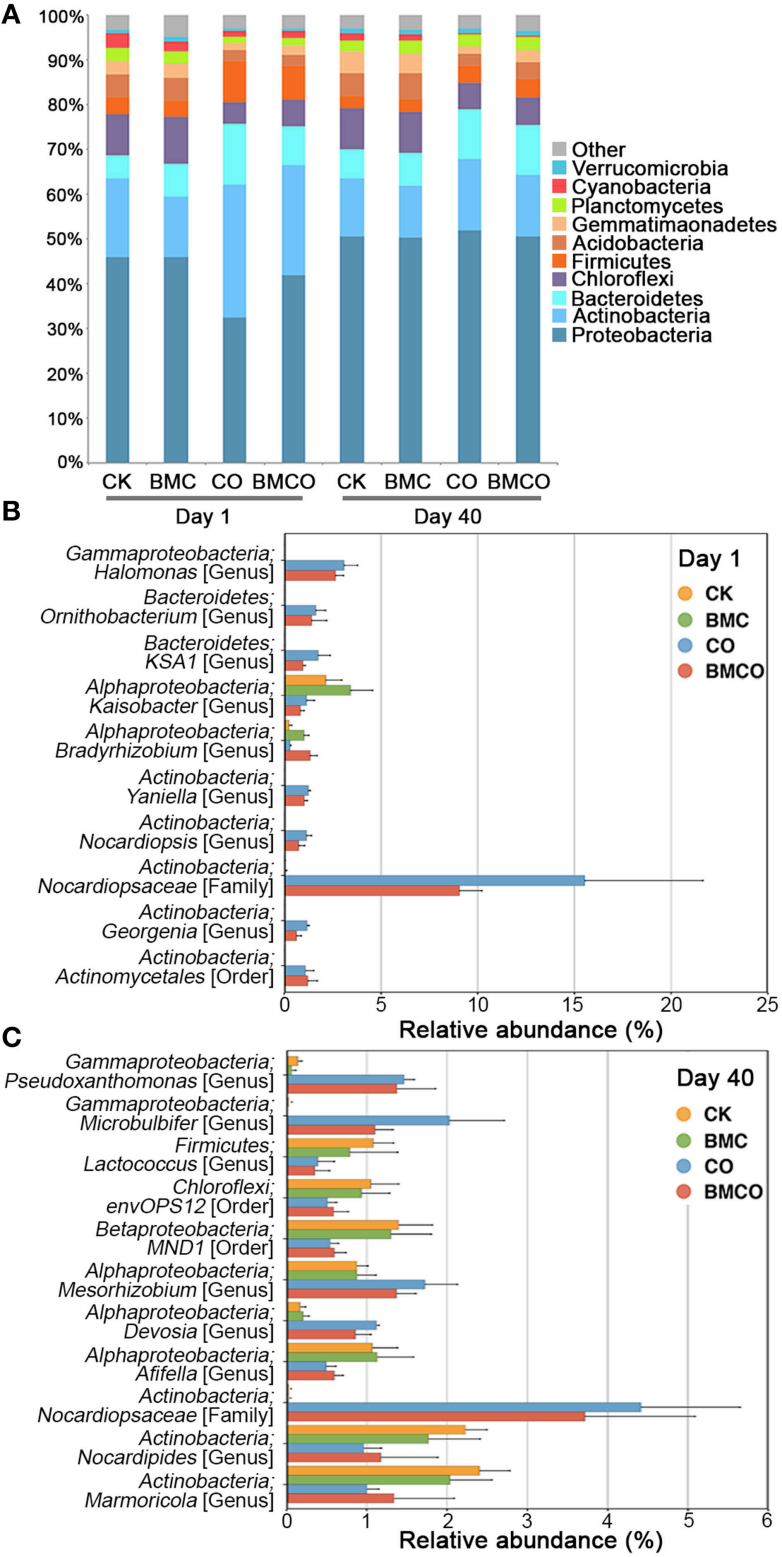
**Bacterial community compositions. (A)** The distribution of the 10 most abundant phyla across treatments in day 1 and day 40. **(B)** The average relative abundance of 10 most abundant OTUs that significantly differed in comparison of treatments with and without compost (CK and BMC vs. CO and BMCO) in day 1, *n* = 5. **(C)** The average relative abundance of 10 most abundant OTUs that significantly differed in comparison of treatments with and without compost (CK and BMC vs. CO and BMCO) in day 40, *n* = 5. *P* < 0.05 with adjustment of Benjamini–Hochberg for multiple comparisons.

Given the clear distinction of bacterial communities between treatments and their correlation with edaphic parameters, we next explored which specific microbial taxa were present and responsive in each treatment. Proteobacteria, Actinobacteria, and Bacteroidetes were the three most dominant phyla (Figure 2A), which accounted for 73.9% of bacterial diversity seen across all samples. The phyla Actinobacteria, Bacteroidetes, and Firmicutes were more abundant at day 1 in the treatment with compost (CO and BMCO) compared to the treatments without compost (CK and BMC). After 40 days, phylum composition became more similar across treatments (Figure 2A), which was consistent with the measurements of α-diversity (see Table S2). Larger compositional changes during the 40 days pot trial were found in the CO treatment compared with CK at the phylum level, with the CK treatment exhibited 92.4% similarity and the CO treatment having only 76.9% between the beginning and end of the pot trial. This suggests that the treatments with compost had a relatively large turnover in the bacterial communities compared to unamended soil.

We next analyzed OTU-based community profiles from day 1 and 40 separately to further define differences between treatments. At the beginning of the pot trial, the application of compost introduced 59 new OTUs into the soil, which mainly belonged to the phyla Actinobacteria (49.2%), Proteobacteria (22.8%), Bacteroidetes (16.5%), and Firmicutes (9.5%). This introduction of new bacteria was further illustrated when only the 10 most abundant OTUs were considered (see Figure 2B). For example, an OTU assigned to the family *Nocardiopsaceae* (phylum Actinobacteria) made up between 9 and 15% of the bacterial communities in treatments receiving compost, but was virtually absent in soil without amendment. After 40 days, however, other OTUs contributed most strongly to the difference between treatments, including those belonging to the genera *Pseudoxanthomonas, Microbulbifer, Mesorhizobium*, and *Devosia* (Figure 2C). The treatment BMCO showed significant increase in OTUs assigned to the taxa *Ellin6067, MND1, Ellin6075*, and *Cytophagaceae* compared to the treatment CO (Figure S2).

**Figure 3:**
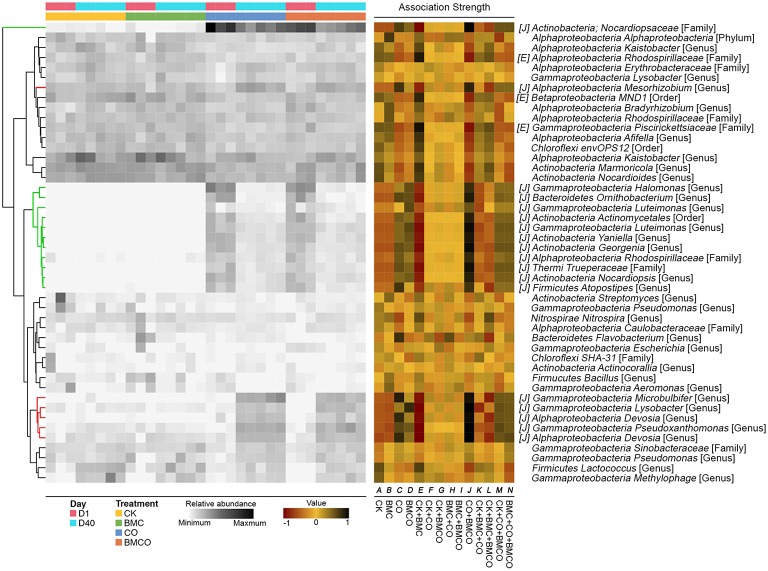
**Bacterial operational taxonomic units (OTUs) identified by indicator analysis as indicative species of each treatment or the combination of treatments**. The heatmap in the left-hand portion of the panel shows relative abundance (>1% in at least one sample) of OTUs across all samples. OTUs are clustered using unsupervised hierarchical clustering based on relative abundance. The green portion of the tree encompasses OTUs that are introduced by compost whereas the red portion encompasses soil indigenous OTUs that are stimulated by compost. Association strength is presented in the middle portion of the panel, with “strength” for a given OTU defined as its preference for corresponding treatment or combination of treatments (−1, avoiding the site; +1, prevailing in the site). The letters in square brackets denotes OTUs that had significant association strength with corresponding treatment or combination of treatments (*P*_*adjust*_ < 0.05). The OTU names are formatted as: *name of class level*; *name of the lowest classified level* [the lowest classified level]. A full list of indicative OTUs is provided in Table S4.

Indicator species analysis further identified 144 treatment-associated OTUs, which accounted for 21.2% of all sequences. There were 7, 10, 18, and 3 OTUs to be associated with treatments of CK, BMC, CO, and BMCO, respectively (Figure 3, and Table S4 for details). No OTUs were associated with combinations between CK and CO treatments indicating the distinct biotic environment of these two treatments. Furthermore, many OTUs were introduced into soil through the compost, but depleted (or even disappeared) over time, while several indigenous OTUs were stimulated in the treatments CO and BMCO (Figure 3).

## Discussion

In this study we aimed to define the effect that compost and biochar–mineral complex (BMC) have on the bacterial communities in soil and on the cultivation of pakchoi. Overall, the results show that soil, plants, and bacterial communities were clearly influenced by the amendment of compost or biochar–mineral complex, either alone or in combination (Figure 1). Compost was the main factor shaping the soil environment, plant growth and microbiota. Specifically, compost added both high levels of nutrients and distinct microorganisms to soil, but the relative abundance of introduced bacteria were reduced after 40 days of cultivation and instead other soil-borne microorganisms dominated the community (Figures 2, 3). BMC was shown here to interact synergistically with compost, both in terms of certain edaphic parameters (i.e. TC, TN, OC, ${\text{NO}}_{3}^{-}$, and AK) and plant properties (i.e., leaf area and nitrate levels, Table 3). We also identified a range of strong treatment-associated taxa that might play key roles in mediating positive outcomes for the soil and plant environment.

### The impact of compost on nutrient processes in soil

The typical application rate (i.e., 28.5 t ha^−1^; Wong et al., 1999) used here for compost clearly had a major impact on the soil environment. Soil microorganisms are the first responders and degraders of soil nutrients and our analysis showed that substantial changes in the bacterial community structure and composition resulted from the addition of compost (Figure 1C; Figure S1; Table S2). These are consistent with recent studies that show compost to affect soil biodiversity (Mäder et al., 2002; Hartmann et al., 2015). Therefore, compost did not only bring nutrients into the soil environment, but also stimulated the growth and turnover of bacteria in the soil. This outcome would be a transformation of the soil's microbial processes, which could subsequently impact on nutrient levels and plant performance.

We observed significant differences in OTUs assigned to the phyla Chloroflexi, Acidobacteria, Actinobateria, Bacteroidetes, and Proteobacteria between treatments with and without compost (Figures 2, 3). Chloroflexi and Acidobacteria, which have previously been shown to be slow-growing bacteria (Davis et al., 2011) and generally prefer oligotrophic environment (Fierer et al., 2012; Hartmann et al., 2015), were strongly associated with the BMC and CK treatments (Table S4). OTUs that were abundant in CO and BMCO treatments within the phylum Actinobacteria were assigned to the genera *Streptomyces, Phytohabitans, Aeromicrobium, Nocardioides*, and *Pimelobacter*, which are known to be lignocellulose decomposers and play important roles in organic matter turnover (Abdulla and El-Shatoury, 2007). Moreover, OTUs within the families *Cytophagaceae* and *Chitinophagaceae* (phylum Bacteroidetes), which are known to degrade complex carbohydrates (McBride et al., 2014; Rosenberg, 2014), were associated with compost (Table S4). The OTUs stimulated by compost in the class *Gammaproteobacteria* belonged to the genera *Microbulbifer, Lysobacter*, and *Pseudoxanthomonas* (Figure 3). *Microbulbifer* spp. excrete hydrolytic enzymes for the breakdown of cellulose, chitin and gelatin (González et al., 1997). *Lysobacter* spp. are well-known biocontrol agents, producing several antibacterial natural products (Xie et al., 2012). *Pseudoxanthomonas* spp. have been shown to degrade various organic compounds (Kim et al., 2008).

Among the *Alphaproteobacteria*, a total of 14 treatment-associated OTUs for the four treatments were classified to the order *Rhizobiales* and they have distinct preferences for certain treatments and metabolic processes. Among the six OTUs that were enriched in CK and BMC treatments, five were assigned to the genera *Rhodoplanes, Hyphomicrobium*, and *Pedomicrobium*m, which are known to be chemoorganotrophic. Members of these genera usually utilize simple carbon substrates and mineral salts and are adapted to nutrient-poor habitats (Oren and Xu, 2014). Furthermore, *Pedomicrobium* spp. and *Hyphomicrobium* spp. are known as prosthecate bacteria that have selective advantage in oligotrophic environments (Semenov and Staley, 1992). In contrast, the eight OTUs associated with CO and BMCO treatments were assigned to the genera *Devosia, Mesorhizobium, Agrobacterium*, and *Shinella*. The chemoheterotrophic *Devosia* spp. possess genes for nitrogen fixation and nodulation (Rivas et al., 2002; Bautista et al., 2010), while the genus *Shinella* has been found in copiotrophic environment (Alves et al., 2014), including root nodules of herbal legumes (Lin et al., 2008). *Mesorhizobium* spp. and *Agrobacterium* spp. are well-known diazotrophs (Lippincott et al., 1981) and have plant growth-promoting abilities (Hao et al., 2012).

Together these information demonstrate that unamended soil and soil amended with low amounts (1.5 t ha^−1^) of relatively recalcitrant BMC maintained bacterial communities that are oligotrophic in nature. This indicates that the natural soil used for pakchoi cultivation was nutrient-limited, which was also reflected in the poor plant yields and high soluble saccharides (Roitsch, 1999). Addition of compost, regardless of BMC being present or not, overcomes this restrictions and facilitates the growth of many bacteria, that are not only adapted to more copiotrophic conditions, but also have features, such as nitrogen fixation, that are beneficial for the growth of pakchoi. As a consequence the increased yield seen here was likely not only an outcome of additional nutrients, but also due to stimulation of beneficial microbial organisms and processes.

In addition, an OTU assigned to the *Nocardiopsaceae* was the most abundant taxa introduced by compost and accounted for 15.52 ± 6.12 and 9.05 ± 1.17% of the total relative abundance in the CO and BMCO treatments at day 1, respectively. Its abundance decreased substantially during the 40 days of cultivation (4.1 ± 1.23 and 3.72 ± 1.37% for CO and BMCO treatments, respectively). A representative sequence for this *Nocardiopsaceae* OTU showed a 100% identity to the 16S rRNA gene sequence of an isolate from cattle skin lesions (Genbank accession JQ031766) and 96% identity to the 16S rRNA gene of *Nocardiopsis nikkonensis*, which was isolated from matured compost (Yamamura et al., 2010). There were four additional OTUs assigned to the genera *Yaniella, Georgenia, Halomonas*, and *Ornithobacterium* introduced by compost, but that decreased significantly by day 40. Representative sequences of the *Yaniella, Georgenia*, and *Halomonas* OTUs were found to be most similar to cultured strains from halophilic environments (Vreeland et al., 1980; Li et al., 2004; Tang et al., 2010). The *Ornithobacterium* OTU was similar to strains of fatal pathogen for poultry (Hafez, 2002). This shows that compost delivered a diverse range of microorganisms into soil, including potential pathogens from the animal sources. However, these microorganisms derived from the compost feedstock (i.e., chicken manure) decreased in abundance, which is likely due to being exposed to the different environmental conditions (such as temperature, humidity, salinity or nutrient availability) present the soil (Sun et al., 2015).

### Additive and synergistic effects of biochar–mineral complex with compost

BMC on its own was not sufficient to achieve the same plant yields as the CO treatment, but we found a number of additive and synergistic effects when BMC and compost were combined. Of particular interest is that the BMCO treatment had increased values for TC, TN, OC, TSN, ${\text{NO}}_{3}^{-}$, and AK, often beyond the level expected by simply adding the separate effects of CO and BMC. For example, the level of ${\text{NO}}_{3}^{-}$ in the BMCO treatment was two times higher (8.81 ± 2.34 mg kg^−1^) than what was obtained by the CO treatment (4.02 mg ± 1.25 kg^−1^) and could not be explained by simply accounting for the effect of BMC alone (3.08 ± 0.75 mg kg^−1^) and the soil control (1.96 ± 0.53 mg kg^−1^). Soil nitrate is an important factor for plant growth and has been shown to stimulate the growth of leaves (Guo et al., 2007). Thus, we propose that the increased leaf size seen in our pakchoi experiment was a direct consequence of increased nitrate level caused by synergistic activities between BMC and compost. Likewise, we found that the net increment of TC and TN in the CO and BMCO treatments compared to the CK were on average three and two times greater than could be explained by the carbon and nitrogen added through the compost and BMC plus compost (Tables 1, 2). These increase can potentially be explained by the enrichment of certain types of bacteria, such as decomposing (e.g., *Streptomyces* spp. or *Cytophagaceae*) or nitrogen-fixing (e.g., *Mesorhizobium* spp. or *Agrobacterium* spp.) taxa (see above). While this increase in the BMCO treatment had no immediate benefits for the plant growth in the short time frame of our growth experiment, we expect that longer trials, in particular those with multiple growing seasons, will see a continued increase in soil nutrients and thus likely sustained productivity.

One explanation for the increased nitrate values seen in the BMCO treatments is in the underlying microbial processes. We found that OTUs of the taxa *Ellin6067, MND1*, and *Ellin6075* were significantly more abundant in BMCO treatments when compared with the CO at day 40 (Figure S2). *Ellin6067* spp. has been reported as a putative ammonia-oxidizing bacterium (Xia et al., 2005) and *MND1* spp. as well as *Ellin6075* spp. are capable of nitrification (Chuang et al., 2007; Cao et al., 2015). This implies that nitrification processes were increased in the BMCO treatment. We further investigated this by summing up all known nitrifying bacteria (using the keyword “nitro” in the taxonomic name). At day 1 the soil contained a relative abundance of 0.78 ± 0.11% for these summed nitrifying bacteria. After 40 days, BMC had a significant effect on the relative abundance of nitrifying bacteria (*P* = 0.007) with the BMCO treatment having the highest number of nitrifying bacteria (1.10 ± 0.07%) followed by the BMC (1.04 ± 0.04%), CK (0.95 ± 0.09%), and CO (0.79 ± 0.05%) treatments. Increased nitrifying activities, as indicated by a relative higher number in nitrifying bacteria, would result in higher nitrate levels (Figure 3) and this in turn could result in the increase of pakchoi leaf size. We therefore propose that, in our case, the increased pakchoi leaf areas was an indirect consequence of BMC stimulating nitrification in soils that have been amended with compost.

We also observed that the content of available phosphate decreased in the BMCO treatment relative to the CO treatment (Table 3). Orthophosphate (the main form of available phosphate) is the second most limiting macronutrient (after nitrogen) for plant growth (Schachtman et al., 1998) and crops have a high requirement of phosphate fertilizers when grown on slightly alkaline soil (Lucas and Davis, 1961). Therefore, it is likely that the relatively high pH of compost and the metal ions (Ca^2+^, Mg^2+^, Fe^3+^, Al^3+^) on BMC (Table 1) provided conditions to immobilize orthophosphate leading to low amounts of available phosphate and hence limiting plant growth (Cao and Harris, 2010). However, this effect might only be short term, as a hardwood biochar, which has similar properties to the biochar used here except for the mineral coating, was found to promote the growth of phosphorus-mobilizing bacteria and subsequently the growth of ryegrass *Lolium perenne* (Fox et al., 2014). As our pot trial lasted only 40 days such positive effects of microbial phosphate solubilization might not have had enough time to develop.

## Conclusion

This study provides evidence that BMC can act synergistically with compost to mediate microbial processes that result in changes in soil nutrient cycles, which in turn can impact onto agricultural properties. Based on our observation we propose a model, whereby the large amount of nutrients afforded by compost accelerates soil processes, such as nutrient and bacterial community turnover. These processes can then be controlled (or steered) by the addition of BMC, whose intricate chemical properties and surface structures are known to influence the chemico-physical properties of the soil environment (e.g., humidity, Figure S1) as well as microbial metabolism and diversity (Joseph et al., 2013). Designing BMCs in the future to match particular organic fertilizer amendments and soil parameters could thus produce microbially mediated nutrient conversions with improved agricultural productivity. BMC-compost combinations that have reduced amounts of microbially mediated N_2_O and methane emission could also be potentially designed, similar to what has been recently observed for the addition of plain biochar to agricultural soils (Harter et al., 2014; Ho et al., 2015).

## Author contributions

JY, RZ, SN, SDJ, DH, and TT conceived and designed the research; JY and RZ performed the research; JY, RZ, SN, DH, and TT analyzed data; JY and TT wrote the paper.

### Conflict of interest statement

The authors declare that the research was conducted in the absence of any commercial or financial relationships that could be construed as a potential conflict of interest.
