# Supplementary material for: A Combination of Biochar–Mineral Complexes and Compost Improves Soil Bacterial Processes, Soil Quality, and Plant Properties
Source: Front Microbiol. 2016 Apr 8;7:372. doi: 10.3389/fmicb.2016.00372 (PMC4824760; doi:10.3389/fmicb.2016.00372)
Supplement: Supplementary file 1 [file Table1.docx]

**Supplementary Material**

**A combination of biochar-mineral complexes and compost improves soil bacterial processes, soil quality and plant properties**

Jun Ye^1,§^, Rui Zhang^2,§^, Shaun Nielsen^1^, Stephen D. Joseph^3^, Danfeng Huang^2*^, Torsten Thomas^1*^

*1. Centre for Marine Bio-Innovation & School of Biotechnology and Biomolecular Sciences, The University of New South Wales, Sydney, Australia*

*2. School of Agriculture and Biology, Shanghai Jiaotong University, Shanghai, China 3. School of Materials Science and Engineering, The University of New South Wales, Sydney, Australia*

*^§^ These authors contributed equally to this work*

^*^ Corresponding authors:

**Supplementary Tables and Figures:**

**Table S1** Effect of factors (i.e. CO; BMC) and their interaction (i.e. OF × BMC) on soil and plant variables assessed by analysis of variance (ANOVA). Degrees of freedom and denominator are given in brackets (*F_(1, 16)_*). Values at *P* < 0.05 are shown in bold.

**Table S2** Effect of factors (i.e. CO, compost; BMC, biochar-mineral complex; CT, cultivation time) and their interactions (i.e. CO × BMC; CO × CT; BMC × CT; CO × BMC × CT) on bacterial alpha diversity assessed by analysis of variance (ANOVA). Degrees of freedom and denominator are given in brackets (*F_(1, 25)_*). Due to the significant interaction between CO and CT on bacterial evenness, pairwise test (CO : CT) was applied within the levels of CT (D1, day 1; D40, day 40) to test the effect of CO (Y, with; N, without) separately. Values at *P* < 0.05 are shown in bold. Average evenness and richness (mean ± s.e.; n = 3 in day 1, n = 5 in day 40) for each treatments are shown. Low case letters represent significant differences at *P* < 0.05.

**Table S3** Effect of factors (i.e. CO, compost; BMC, biochar-mineral complex; CT, cultivation time) and pairwise test on dispersion assessed by permutational homogeneity of dispersion test (PERMDISP). Values represent the pseudo-*F* ratio with degrees of freedom and denominator in brackets (*F_(1, 31)_*), the permutation-based level of significance (*P*), the pairwise t-statistic (*t*). Values at *P* < 0.05 are shown in bold. The average distances to group centroid of treatments are shown (mean ± s.e.; n = 3 in day 1, n = 5 in day 40). D1 represent all samples in day 1, D40 represent all samples in day 40.

|  | Kingdom | Phylum | Class | Order | Family | Genus | AS | *P_adjust_* | CK | BMC | CO | BMCO |
| --- | --- | --- | --- | --- | --- | --- | --- | --- | --- | --- | --- | --- |
| Otu00145 | Bacteria | Proteobacteria | Betaproteobacteria | Methylophilales | Methylophilaceae | unclassified | 0.836 | 0.012 | 1 | 0 | 0 | 0 |
| Otu00393 | Bacteria | Acidobacteria | Chloracidobacteria | RB41 | unclassified | unclassified | 0.704 | 0.046 | 1 | 0 | 0 | 0 |
| Otu00396 | Bacteria | Proteobacteria | Gammaproteobacteria | Pseudomonadales | Moraxellaceae | Enhydrobacter | 0.714 | 0.041 | 1 | 0 | 0 | 0 |
| Otu00686 | Bacteria | Gemmatimonadetes | Gemmatimonadetes | Ellin5290 | unclassified | unclassified | 0.808 | 0.026 | 1 | 0 | 0 | 0 |
| Otu00853 | Bacteria | Firmicutes | Clostridia | Clostridiales | Clostridiaceae | unclassified | 0.693 | 0.047 | 1 | 0 | 0 | 0 |
| Otu00956 | Bacteria | Gemmatimonadetes | Gemm-5 | unclassified | unclassified | unclassified | 0.768 | 0.044 | 1 | 0 | 0 | 0 |
| Otu01682 | Bacteria | Firmicutes | Bacilli | Bacillales | Alicyclobacillaceae | Alicyclobacillus | 0.741 | 0.039 | 1 | 0 | 0 | 0 |
| Otu01315 | Bacteria | Proteobacteria | Alphaproteobacteria | Rhodospirillales | Rhodospirillaceae | unclassified | 0.952 | 0.010 | 0 | 1 | 0 | 0 |
| Otu00573 | Bacteria | Actinobacteria | Actinobacteria | Micromonosporales | Micromonosporaceae | Phytohabitans | 0.849 | 0.023 | 0 | 1 | 0 | 0 |
| Otu01380 | Bacteria | WS3 | PRR-12 | Sediment-1 | PRR-10 | unclassified | 0.846 | 0.028 | 0 | 1 | 0 | 0 |
| Otu00137 | Bacteria | Bacteroidetes | Cytophagia | Cytophagales | Cytophagaceae | unclassified | 0.817 | 0.014 | 0 | 1 | 0 | 0 |
| Otu00178 | Bacteria | Acidobacteria | Acidobacteria-5 | unclassified | unclassified | unclassified | 0.787 | 0.030 | 0 | 1 | 0 | 0 |
| Otu00519 | Bacteria | Bacteroidetes | Saprospirae | Saprospirales | Chitinophagaceae | unclassified | 0.786 | 0.030 | 0 | 1 | 0 | 0 |
| Otu00279 | Bacteria | Proteobacteria | Alphaproteobacteria | Rhizobiales | unclassified | unclassified | 0.762 | 0.041 | 0 | 1 | 0 | 0 |
| Otu00049 | Bacteria | Proteobacteria | Alphaproteobacteria | Rhizobiales | Hyphomicrobiaceae | Rhodoplanes | 0.762 | 0.032 | 0 | 1 | 0 | 0 |
| Otu00885 | Bacteria | Proteobacteria | Alphaproteobacteria | Rhizobiales | Hyphomicrobiaceae | Hyphomicrobium | 0.730 | 0.038 | 0 | 1 | 0 | 0 |
| Otu00553 | Bacteria | Verrucomicrobia | Opitutae | Cerasicoccales | Cerasicoccaceae | unclassified | 0.641 | 0.038 | 0 | 1 | 0 | 0 |
| Otu00156 | Bacteria | Proteobacteria | Betaproteobacteria | Burkholderiales | Oxalobacteraceae | Duganella | 0.758 | 0.030 | 0 | 0 | 1 | 0 |
| Otu00180 | Bacteria | Proteobacteria | Gammaproteobacteria | Alteromonadales | Alteromonadaceae | Cellvibrio | 0.890 | 0.010 | 0 | 0 | 1 | 0 |
| Otu00242 | Bacteria | Bacteroidetes | Flavobacteriia | Flavobacteriales | Flavobacteriaceae | Flavobacterium | 0.780 | 0.030 | 0 | 0 | 1 | 0 |
| Otu00425 | Bacteria | Bacteroidetes | Flavobacteriia | Flavobacteriales | Flavobacteriaceae | Flavobacterium | 0.732 | 0.026 | 0 | 0 | 1 | 0 |
| Otu00466 | Bacteria | Proteobacteria | Alphaproteobacteria | Rhizobiales | Hyphomicrobiaceae | Devosia | 0.728 | 0.047 | 0 | 0 | 1 | 0 |
| Otu00604 | Bacteria | Actinobacteria | Actinobacteria | Actinomycetales | Streptomycetaceae | Streptomyces | 0.683 | 0.039 | 0 | 0 | 1 | 0 |
| Otu00655 | Bacteria | Actinobacteria | Actinobacteria | Actinomycetales | Mycobacteriaceae | Mycobacterium | 0.763 | 0.030 | 0 | 0 | 1 | 0 |
| Otu00662 | Bacteria | Bacteroidetes | Flavobacteriia | Flavobacteriales | Flavobacteriaceae | Flavobacterium | 0.848 | 0.010 | 0 | 0 | 1 | 0 |
| Otu00672 | Bacteria | Bacteroidetes | Cytophagia | Cytophagales | Cytophagaceae | unclassified | 0.833 | 0.026 | 0 | 0 | 1 | 0 |
| Otu00727 | Bacteria | Bacteroidetes | Flavobacteriia | Flavobacteriales | Cryomorphaceae | Fluviicola | 0.886 | 0.010 | 0 | 0 | 1 | 0 |
| Otu00736 | Bacteria | Proteobacteria | Deltaproteobacteria | Myxococcales | OM27 | unclassified | 0.753 | 0.038 | 0 | 0 | 1 | 0 |
| Otu00742 | Bacteria | NKB19 | TSBW08 | unclassified | unclassified | unclassified | 0.780 | 0.032 | 0 | 0 | 1 | 0 |
| Otu00886 | Bacteria | Proteobacteria | Betaproteobacteria | unclassified | unclassified | unclassified | 0.851 | 0.026 | 0 | 0 | 1 | 0 |
| Otu00936 | Bacteria | Actinobacteria | Actinobacteria | Propionibacteriales | Nocardioidaceae | Pimelobacter | 0.763 | 0.047 | 0 | 0 | 1 | 0 |
| Otu01094 | Bacteria | Proteobacteria | Gammaproteobacteria | Oceanospirillales | Hahellaceae | Hahella | 0.874 | 0.026 | 0 | 0 | 1 | 0 |
| Otu01294 | Bacteria | Firmicutes | Bacilli | Lactobacillales | Lactobacillaceae | Lactobacillus | 0.803 | 0.038 | 0 | 0 | 1 | 0 |
| Otu01485 | Bacteria | Actinobacteria | Acidimicrobiia | Acidimicrobiales | AKIW874 | unclassified | 0.794 | 0.040 | 0 | 0 | 1 | 0 |
| Otu01507 | Bacteria | Bacteroidetes | Cytophagia | Cytophagales | Cytophagaceae | Adhaeribacter | 0.908 | 0.010 | 0 | 0 | 1 | 0 |
| Otu00467 | Bacteria | Firmicutes | Clostridia | Clostridiales | Lachnospiraceae | unclassified | 0.807 | 0.030 | 0 | 0 | 0 | 1 |
| Otu02354 | Bacteria | Bacteroidetes | Saprospirae | Saprospirales | Chitinophagaceae | Flavisolibacter | 0.835 | 0.030 | 0 | 0 | 0 | 1 |
| Otu02361 | Bacteria | Planctomycetes | Phycisphaerae | WD2101 | unclassified | unclassified | 0.819 | 0.038 | 0 | 0 | 0 | 1 |
| Otu00005 | Bacteria | Proteobacteria | Betaproteobacteria | MND1 | unclassified | unclassified | 0.740 | 0.042 | 1 | 1 | 0 | 0 |
| Otu00011 | Bacteria | Proteobacteria | Gammaproteobacteria | Thiotrichales | Piscirickettsiaceae | unclassified | 0.840 | 0.010 | 1 | 1 | 0 | 0 |
| Otu00018 | Bacteria | Proteobacteria | Alphaproteobacteria | Rhodospirillales | Rhodospirillaceae | unclassified | 0.803 | 0.027 | 1 | 1 | 0 | 0 |
| Otu00030 | Bacteria | Proteobacteria | Alphaproteobacteria | Rhizobiales | Hyphomicrobiaceae | Pedomicrobium | 0.726 | 0.026 | 1 | 1 | 0 | 0 |
| Otu00035 | Bacteria | Proteobacteria | Alphaproteobacteria | Rhizobiales | Hyphomicrobiaceae | Rhodoplanes | 0.709 | 0.050 | 1 | 1 | 0 | 0 |
| Otu00050 | Bacteria | Gemmatimonadetes | Gemm-1 | unclassified | unclassified | unclassified | 0.734 | 0.042 | 1 | 1 | 0 | 0 |
| Otu00058 | Bacteria | Proteobacteria | Betaproteobacteria | MND1 | unclassified | unclassified | 0.754 | 0.037 | 1 | 1 | 0 | 0 |
| Otu00066 | Bacteria | Gemmatimonadetes | Gemm-5 | unclassified | unclassified | unclassified | 0.768 | 0.030 | 1 | 1 | 0 | 0 |
| Otu00067 | Bacteria | Proteobacteria | Alphaproteobacteria | Rhizobiales | Hyphomicrobiaceae | Rhodoplanes | 0.806 | 0.010 | 1 | 1 | 0 | 0 |
| Otu00089 | Bacteria | Gemmatimonadetes | Gemmatimonadetes | unclassified | unclassified | unclassified | 0.800 | 0.017 | 1 | 1 | 0 | 0 |
| Otu00094 | Bacteria | Acidobacteria | Acidobacteria-6 | iii1-15 | unclassified | unclassified | 0.795 | 0.002 | 1 | 1 | 0 | 0 |
| Otu00111 | Bacteria | Proteobacteria | Gammaproteobacteria | Pseudomonadales | Pseudomonadaceae | Pseudomonas | 0.752 | 0.026 | 1 | 1 | 0 | 0 |
| Otu00122 | Bacteria | Proteobacteria | Gammaproteobacteria | Xanthomonadales | Sinobacteraceae | unclassified | 0.775 | 0.038 | 1 | 1 | 0 | 0 |
| Otu00255 | Bacteria | Chloroflexi | Anaerolineae | DRC31 | unclassified | unclassified | 0.747 | 0.048 | 1 | 1 | 0 | 0 |
| Otu00257 | Bacteria | Gemmatimonadetes | Gemmatimonadetes | N1423WL | unclassified | unclassified | 0.744 | 0.025 | 1 | 1 | 0 | 0 |
| Otu00382 | Bacteria | Acidobacteria | iii1-8 | DS-18 | unclassified | unclassified | 0.695 | 0.045 | 1 | 1 | 0 | 0 |
| Otu00505 | Bacteria | Gemmatimonadetes | Gemmatimonadetes | unclassified | unclassified | unclassified | 0.726 | 0.045 | 1 | 1 | 0 | 0 |
| Otu00977 | Bacteria | Cyanobacteria | Synechococcophycideae | Pseudanabaenales | Pseudanabaenaceae | Nodosilinea | 0.759 | 0.039 | 1 | 1 | 0 | 0 |
| Otu01120 | Bacteria | Acidobacteria | Acidobacteria-6 | CCU21 | unclassified | unclassified | 0.784 | 0.033 | 1 | 1 | 0 | 0 |
| Otu01164 | Bacteria | Bacteroidetes | Cytophagia | Cytophagales | Cytophagaceae | unclassified | 0.757 | 0.039 | 1 | 1 | 0 | 0 |
| Otu00001 | Bacteria | Actinobacteria | Actinobacteria | Streptosporangiales | Nocardiopsaceae | unclassified | 0.901 | 0.002 | 0 | 0 | 1 | 1 |
| Otu00006 | Bacteria | Proteobacteria | Alphaproteobacteria | Rhizobiales | Phyllobacteriaceae | Mesorhizobium | 0.750 | 0.031 | 0 | 0 | 1 | 1 |
| Otu00015 | Bacteria | Proteobacteria | Gammaproteobacteria | Oceanospirillales | Halomonadaceae | Halomonas | 0.699 | 0.048 | 0 | 0 | 1 | 1 |
| Otu00016 | Bacteria | Proteobacteria | Gammaproteobacteria | Xanthomonadales | Xanthomonadaceae | Pseudoxanthomonas | 0.930 | 0.002 | 0 | 0 | 1 | 1 |
| Otu00017 | Bacteria | Proteobacteria | Gammaproteobacteria | Alteromonadales | Alteromonadaceae | Microbulbifer | 0.843 | 0.002 | 0 | 0 | 1 | 1 |
| Otu00023 | Bacteria | Proteobacteria | Alphaproteobacteria | Rhizobiales | Hyphomicrobiaceae | Devosia | 0.944 | 0.002 | 0 | 0 | 1 | 1 |
| Otu00024 | Bacteria | Firmicutes | Bacilli | Turicibacterales | Turicibacteraceae | Turicibacter | 0.917 | 0.002 | 0 | 0 | 1 | 1 |
| Otu00037 | Bacteria | Bacteroidetes | Flavobacteriia | Flavobacteriales | Weeksellaceae | Ornithobacterium | 0.799 | 0.002 | 0 | 0 | 1 | 1 |
| Otu00040 | Bacteria | Proteobacteria | Gammaproteobacteria | Xanthomonadales | Xanthomonadaceae | Lysobacter | 0.909 | 0.002 | 0 | 0 | 1 | 1 |
| Otu00041 | Bacteria | Firmicutes | Bacilli | Bacillales | unclassified | unclassified | 0.896 | 0.002 | 0 | 0 | 1 | 1 |
| Otu00044 | Bacteria | Actinobacteria | Actinobacteria | Actinomycetales | Micrococcaceae | Yaniella | 0.825 | 0.010 | 0 | 0 | 1 | 1 |
| Otu00046 | Bacteria | Firmicutes | Clostridia | Clostridiales | Clostridiaceae | SMB53 | 0.821 | 0.012 | 0 | 0 | 1 | 1 |
| Otu00054 | Bacteria | Actinobacteria | Actinobacteria | Actinomycetales | unclassified | unclassified | 0.769 | 0.032 | 0 | 0 | 1 | 1 |
| Otu00057 | Bacteria | Proteobacteria | Alphaproteobacteria | Rhizobiales | Hyphomicrobiaceae | Devosia | 0.878 | 0.002 | 0 | 0 | 1 | 1 |
| Otu00059 | Bacteria | Proteobacteria | Gammaproteobacteria | Xanthomonadales | Xanthomonadaceae | Luteimonas | 0.872 | 0.002 | 0 | 0 | 1 | 1 |
| Otu00063 | Bacteria | Actinobacteria | Actinobacteria | Streptosporangiales | Nocardiopsaceae | Nocardiopsis | 0.788 | 0.032 | 0 | 0 | 1 | 1 |
| Otu00071 | Bacteria | Actinobacteria | Actinobacteria | Micrococcales | Bogoriellaceae | Georgenia | 0.885 | 0.002 | 0 | 0 | 1 | 1 |
| Otu00081 | Bacteria | Proteobacteria | Alphaproteobacteria | Sphingomonadales | Sphingomonadaceae | Novosphingobium | 0.913 | 0.002 | 0 | 0 | 1 | 1 |
| Otu00082 | Bacteria | Proteobacteria | Alphaproteobacteria | Rhizobiales | Hyphomicrobiaceae | Devosia | 0.868 | 0.002 | 0 | 0 | 1 | 1 |
| Otu00084 | Bacteria | Proteobacteria | Gammaproteobacteria | Xanthomonadales | Xanthomonadaceae | unclassified | 0.801 | 0.025 | 0 | 0 | 1 | 1 |
| Otu00085 | Bacteria | Firmicutes | Bacilli | Lactobacillales | Aerococcaceae | Atopostipes | 0.674 | 0.049 | 0 | 0 | 1 | 1 |
| Otu00102 | Bacteria | Proteobacteria | Alphaproteobacteria | Rhodospirillales | Rhodospirillaceae | unclassified | 0.724 | 0.045 | 0 | 0 | 1 | 1 |
| Otu00104 | Bacteria | Proteobacteria | Alphaproteobacteria | Rhizobiales | Rhizobiaceae | Agrobacterium | 0.814 | 0.002 | 0 | 0 | 1 | 1 |
| Otu00109 | Bacteria | Proteobacteria | Deltaproteobacteria | Myxococcales | Nannocystaceae | Nannocystis | 0.869 | 0.002 | 0 | 0 | 1 | 1 |
| Otu00110 | Bacteria | Bacteroidetes | Cytophagia | Cytophagales | Cytophagaceae | unclassified | 0.756 | 0.030 | 0 | 0 | 1 | 1 |
| Otu00113 | Bacteria | Proteobacteria | Alphaproteobacteria | Rhizobiales | Rhizobiaceae | Agrobacterium | 0.830 | 0.004 | 0 | 0 | 1 | 1 |
| Otu00119 | Bacteria | Thermi | Deinococci | Deinococcales | Trueperaceae | B-42 | 0.854 | 0.012 | 0 | 0 | 1 | 1 |
| Otu00128 | Bacteria | Proteobacteria | Gammaproteobacteria | Oceanospirillales | Halomonadaceae | Kushneria | 0.830 | 0.016 | 0 | 0 | 1 | 1 |
| Otu00142 | Bacteria | Firmicutes | Clostridia | Clostridiales | Clostridiaceae | SMB53 | 0.904 | 0.002 | 0 | 0 | 1 | 1 |
| Otu00144 | Bacteria | Proteobacteria | Alphaproteobacteria | Caulobacterales | Caulobacteraceae | Phenylobacterium | 0.882 | 0.002 | 0 | 0 | 1 | 1 |
| Otu00152 | Bacteria | Proteobacteria | Gammaproteobacteria | 34P16 | unclassified | unclassified | 0.750 | 0.031 | 0 | 0 | 1 | 1 |
| Otu00157 | Bacteria | Proteobacteria | Gammaproteobacteria | Xanthomonadales | Xanthomonadaceae | Pseudoxanthomonas | 0.935 | 0.002 | 0 | 0 | 1 | 1 |
| Otu00161 | Bacteria | Proteobacteria | Gammaproteobacteria | Xanthomonadales | Xanthomonadaceae | Dokdonella | 0.778 | 0.025 | 0 | 0 | 1 | 1 |
| Otu00187 | Bacteria | Proteobacteria | Alphaproteobacteria | Caulobacterales | Caulobacteraceae | unclassified | 0.779 | 0.002 | 0 | 0 | 1 | 1 |
| Otu00189 | Bacteria | Proteobacteria | Alphaproteobacteria | Sphingomonadales | Sphingomonadaceae | Novosphingobium | 0.830 | 0.002 | 0 | 0 | 1 | 1 |
| Otu00196 | Bacteria | Proteobacteria | Alphaproteobacteria | Caulobacterales | Caulobacteraceae | Phenylobacterium | 0.753 | 0.026 | 0 | 0 | 1 | 1 |
| Otu00218 | Bacteria | Proteobacteria | Gammaproteobacteria | unclassified | unclassified | unclassified | 0.736 | 0.039 | 0 | 0 | 1 | 1 |
| Otu00220 | Bacteria | Bacteroidetes | Cytophagia | Cytophagales | Cyclobacteriaceae | Algoriphagus | 0.764 | 0.030 | 0 | 0 | 1 | 1 |
| Otu00234 | Bacteria | Bacteroidetes | Cytophagia | Cytophagales | Cytophagaceae | unclassified | 0.883 | 0.002 | 0 | 0 | 1 | 1 |
| Otu00237 | Bacteria | Proteobacteria | Gammaproteobacteria | Xanthomonadales | Xanthomonadaceae | unclassified | 0.815 | 0.010 | 0 | 0 | 1 | 1 |
| Otu00282 | Bacteria | Bacteroidetes | Saprospirae | Saprospirales | Chitinophagaceae | Flavihumibacter | 0.841 | 0.002 | 0 | 0 | 1 | 1 |
| Otu00292 | Bacteria | Fibrobacteres | Fibrobacteria | 258ds10 | unclassified | unclassified | 0.848 | 0.002 | 0 | 0 | 1 | 1 |
| Otu00296 | Bacteria | Bacteroidetes | Cytophagia | Cytophagales | Cytophagaceae | Adhaeribacter | 0.753 | 0.044 | 0 | 0 | 1 | 1 |
| Otu00319 | Bacteria | Proteobacteria | Alphaproteobacteria | Sphingomonadales | Sphingomonadaceae | Novosphingobium | 0.750 | 0.039 | 0 | 0 | 1 | 1 |
| Otu00325 | Bacteria | Firmicutes | Clostridia | Clostridiales | Clostridiaceae | Clostridium | 0.754 | 0.045 | 0 | 0 | 1 | 1 |
| Otu00348 | Bacteria | Firmicutes | Bacilli | Bacillales | Planococcaceae | Sporosarcina | 0.783 | 0.025 | 0 | 0 | 1 | 1 |
| Otu00365 | Bacteria | Actinobacteria | Actinobacteria | Actinomycetales | Microbacteriaceae | Microbacterium | 0.795 | 0.010 | 0 | 0 | 1 | 1 |
| Otu00378 | Bacteria | Proteobacteria | Alphaproteobacteria | Rhizobiales | Rhizobiaceae | Shinella | 0.859 | 0.002 | 0 | 0 | 1 | 1 |
| Otu00405 | Bacteria | Bacteroidetes | Cytophagia | Cytophagales | Cytophagaceae | Sporocytophaga | 0.830 | 0.020 | 0 | 0 | 1 | 1 |
| Otu00408 | Bacteria | Proteobacteria | Alphaproteobacteria | Sphingomonadales | Sphingomonadaceae | unclassified | 0.901 | 0.002 | 0 | 0 | 1 | 1 |
| Otu00436 | Bacteria | Fibrobacteres | Fibrobacteria | 258ds10 | unclassified | unclassified | 0.753 | 0.039 | 0 | 0 | 1 | 1 |
| Otu00443 | Bacteria | Proteobacteria | Alphaproteobacteria | Rhodospirillales | Rhodospirillaceae | Dongia | 0.749 | 0.038 | 0 | 0 | 1 | 1 |
| Otu00455 | Bacteria | Proteobacteria | Gammaproteobacteria | unclassified | unclassified | unclassified | 0.767 | 0.036 | 0 | 0 | 1 | 1 |
| Otu00463 | Bacteria | Proteobacteria | Gammaproteobacteria | Xanthomonadales | Xanthomonadaceae | Thermomonas | 0.793 | 0.026 | 0 | 0 | 1 | 1 |
| Otu00517 | Bacteria | Proteobacteria | Gammaproteobacteria | Xanthomonadales | Xanthomonadaceae | Luteimonas | 0.743 | 0.045 | 0 | 0 | 1 | 1 |
| Otu00518 | Bacteria | Fibrobacteres | Fibrobacteria | 258ds10 | unclassified | unclassified | 0.715 | 0.045 | 0 | 0 | 1 | 1 |
| Otu00521 | Bacteria | Proteobacteria | Alphaproteobacteria | Rickettsiales | mitochondria | unclassified | 0.878 | 0.003 | 0 | 0 | 1 | 1 |
| Otu00523 | Bacteria | Proteobacteria | Gammaproteobacteria | Xanthomonadales | Xanthomonadaceae | Luteimonas | 0.808 | 0.016 | 0 | 0 | 1 | 1 |
| Otu00525 | Bacteria | Proteobacteria | Deltaproteobacteria | Myxococcales | Polyangiaceae | unclassified | 0.799 | 0.021 | 0 | 0 | 1 | 1 |
| Otu00547 | Bacteria | Bacteroidetes | Flavobacteriia | Flavobacteriales | Flavobacteriaceae | Flavobacterium | 0.784 | 0.031 | 0 | 0 | 1 | 1 |
| Otu00549 | Bacteria | Fibrobacteres | Fibrobacteria | 258ds10 | unclassified | unclassified | 0.732 | 0.038 | 0 | 0 | 1 | 1 |
| Otu00568 | Bacteria | Proteobacteria | Deltaproteobacteria | Myxococcales | Polyangiaceae | Sorangium | 0.768 | 0.032 | 0 | 0 | 1 | 1 |
| Otu00576 | Bacteria | Bacteroidetes | Saprospirae | Saprospirales | Chitinophagaceae | Flavihumibacter | 0.816 | 0.010 | 0 | 0 | 1 | 1 |
| Otu00586 | Bacteria | Bacteroidetes | Flavobacteriia | Flavobacteriales | Cryomorphaceae | Fluviicola | 0.803 | 0.024 | 0 | 0 | 1 | 1 |
| Otu00612 | Bacteria | Proteobacteria | Gammaproteobacteria | Xanthomonadales | Xanthomonadaceae | Arenimonas | 0.828 | 0.010 | 0 | 0 | 1 | 1 |
| Otu00623 | Bacteria | Bacteroidetes | Cytophagia | Cytophagales | Cytophagaceae | Dyadobacter | 0.787 | 0.030 | 0 | 0 | 1 | 1 |
| Otu00646 | Bacteria | Firmicutes | Clostridia | Clostridiales | Clostridiaceae | Clostridium | 0.726 | 0.047 | 0 | 0 | 1 | 1 |
| Otu00681 | Bacteria | Actinobacteria | Actinobacteria | Actinomycetales | Microbacteriaceae | Microbacterium | 0.732 | 0.036 | 0 | 0 | 1 | 1 |
| Otu00782 | Bacteria | Proteobacteria | Deltaproteobacteria | Myxococcales | Polyangiaceae | unclassified | 0.793 | 0.023 | 0 | 0 | 1 | 1 |
| Otu00794 | Bacteria | Bacteroidetes | Flavobacteriia | Flavobacteriales | Flavobacteriaceae | Flavobacterium | 0.783 | 0.030 | 0 | 0 | 1 | 1 |
| Otu00831 | Bacteria | Bacteroidetes | Cytophagia | Cytophagales | Cytophagaceae | unclassified | 0.728 | 0.047 | 0 | 0 | 1 | 1 |
| Otu00888 | Bacteria | Proteobacteria | Alphaproteobacteria | Sphingomonadales | Sphingomonadaceae | unclassified | 0.813 | 0.016 | 0 | 0 | 1 | 1 |
| Otu00891 | Bacteria | Bacteroidetes | Cytophagia | Cytophagales | Cytophagaceae | unclassified | 0.780 | 0.028 | 0 | 0 | 1 | 1 |
| Otu00943 | Bacteria | Bacteroidetes | Saprospirae | Saprospirales | Chitinophagaceae | Lacibacter | 0.851 | 0.004 | 0 | 0 | 1 | 1 |
| Otu00954 | Bacteria | Bacteroidetes | Sphingobacteriia | Sphingobacteriales | Sphingobacteriaceae | Pedobacter | 0.762 | 0.038 | 0 | 0 | 1 | 1 |
| Otu00967 | Bacteria | Fibrobacteres | Fibrobacteria | 258ds10 | unclassified | unclassified | 0.793 | 0.031 | 0 | 0 | 1 | 1 |
| Otu01014 | Bacteria | Verrucomicrobia | Opitutae | Opitutales | Opitutaceae | Opitutus | 0.790 | 0.026 | 0 | 0 | 1 | 1 |
| Otu01080 | Bacteria | Proteobacteria | Deltaproteobacteria | Myxococcales | unclassified | unclassified | 0.784 | 0.029 | 0 | 0 | 1 | 1 |
| Otu01093 | Bacteria | Bacteroidetes | unclassified | unclassified | unclassified | unclassified | 0.749 | 0.044 | 0 | 0 | 1 | 1 |
| Otu01119 | Bacteria | Bacteroidetes | Cytophagia | Cytophagales | Cytophagaceae | unclassified | 0.739 | 0.047 | 0 | 0 | 1 | 1 |
| Otu01243 | Bacteria | Bacteroidetes | Saprospirae | Saprospirales | Chitinophagaceae | Chitinophaga | 0.762 | 0.028 | 0 | 0 | 1 | 1 |
| Otu01306 | Bacteria | Bacteroidetes | Saprospirae | Saprospirales | Chitinophagaceae | unclassified | 0.743 | 0.002 | 0 | 0 | 1 | 1 |
| Otu01328 | Bacteria | Bacteroidetes | Saprospirae | Saprospirales | Chitinophagaceae | unclassified | 0.818 | 0.010 | 0 | 0 | 1 | 1 |
| Otu01604 | Bacteria | Bacteroidetes | Saprospirae | Saprospirales | Chitinophagaceae | Chitinophaga | 0.792 | 0.026 | 0 | 0 | 1 | 1 |
| Otu02210 | Bacteria | Actinobacteria | Actinobacteria | Propionibacteriales | Nocardioidaceae | Nocardioides | 0.750 | 0.005 | 0 | 0 | 1 | 1 |
| Otu00211 | Bacteria | Actinobacteria | Actinobacteria | Propionibacteriales | Nocardioidaceae | Aeromicrobium | 0.715 | 0.047 | 0 | 1 | 1 | 0 |

**Table S4** Taxonomic list of OTUs assigned to treatments (CK: control, BMC: biochar-mineral complex, CO: compost, BMCO: BMC+CO) or their combin ations by indicator analysis. Multiple comparison adjustment was applied using Benjamini-Hochberg procedure. “AS” represent association strength, “*P_adjust_*” represent the level of significance. “1” represent assignment to corresponding treatment.


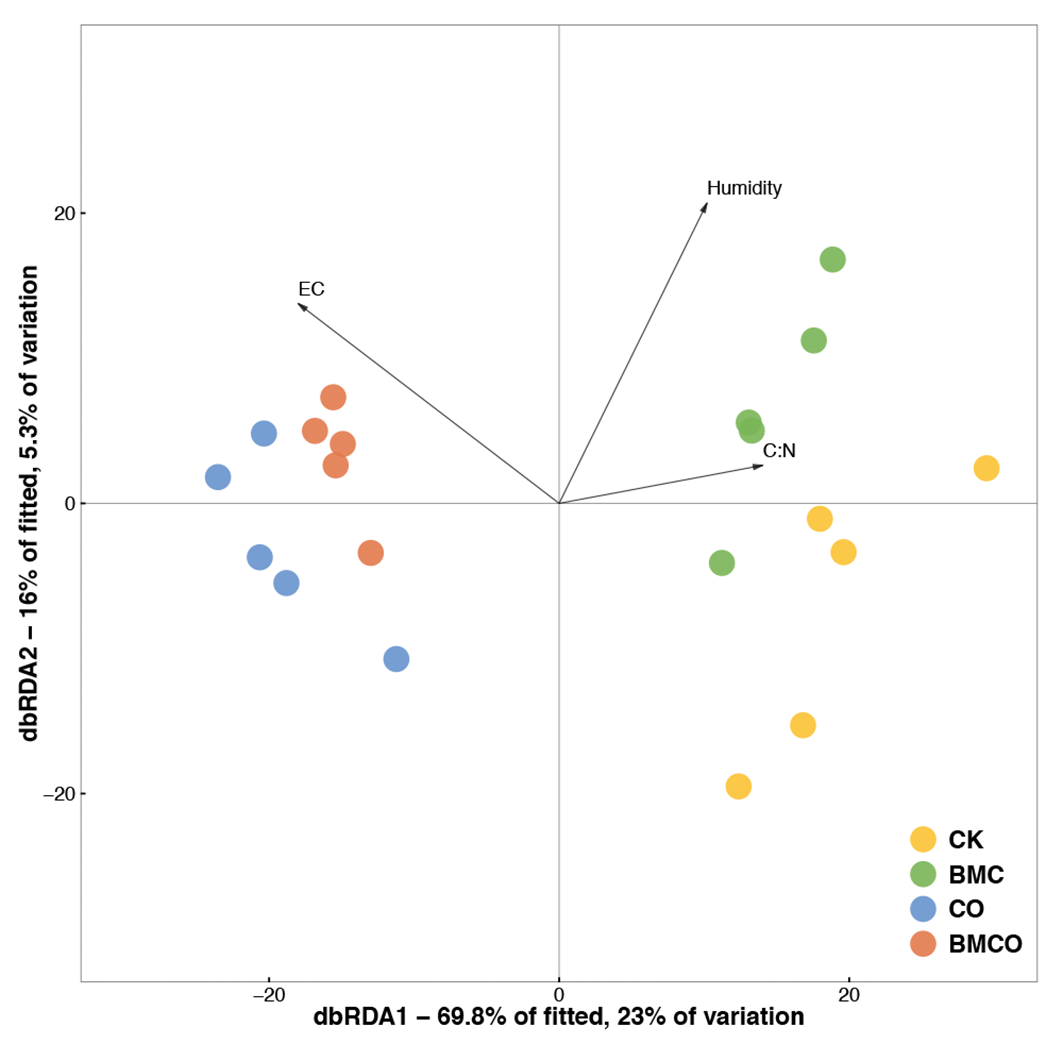


**Figure S1** Distance based redundancy ordination (dbRDA) for the fitted model (edaphic variables) of bacterial communities in day 40 samples based on Bray-Curtis distance matrix.


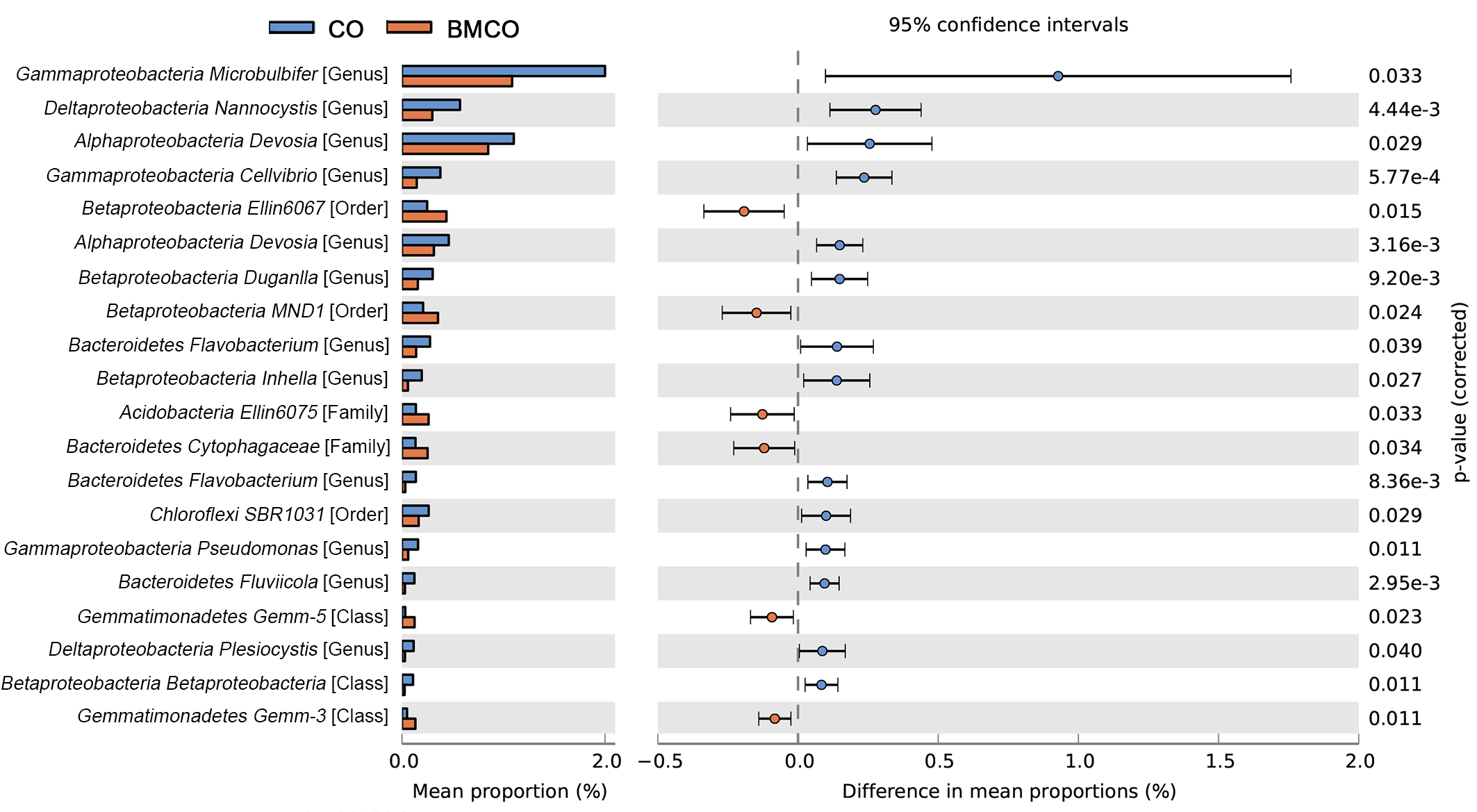


**Figure S2** Extended error bar plot showing the OTUs that have significantly different abundances in BMCO and CO. OTUs overrepresented in the BMCO treatments have a negative difference in relative abundance and OTUs overrepresented in the CO treatment have a positive difference in relative abundance. The first 20 most abundant OTUs that had significant differences between these two treatments are shown.
